# A Text-Book upon the Pathogenic Bacteria

**Published:** 1897-10

**Authors:** 


### Book Notices.

A TEXT-BOOK UPON THE PATHOGENIC BACTERIA—By Joseph McFarland, M. D. 113 illustrations. W. B. Saunders, 925 Walnut Street, Philadelphia. 350 pages. Price, $2.50.

While much is being written on the subject of bacterology the importance of the subject is such that we cannot have too much literature on it provided it is of the right kind. This book will prove especially valuable to the student of bacteriology, since it contains desirable information which cannot be obtained in any other single volume. The technique of bacteriological examinations, with description of culture methods, and the examination of air, water and soil, are given careful consideration. The author discusses immunity, susceptibility, sterilization, disinfection; also the life history of pathogenic bacteria, their culture, appearance, etc. The book is well illustrated, is written in a pleasing style, and altogether is one of the best and most instructive on the subject on which it treats. It is well worthy of a place in any physician's library. H.

